# Incidence of Severe Hepatotoxicity Related to Antiretroviral Therapy in HIV/HCV Coinfected Patients

**DOI:** 10.1155/2010/856542

**Published:** 2010-09-26

**Authors:** Emily L. Heil, Mary L. Townsend, Kenneth Shipp, Amy Clarke, Melissa D. Johnson

**Affiliations:** ^1^Eshelman School of Pharmacy, The University of North Carolina at Chapel Hill, Chapel Hill, NC 27599, USA; ^2^College of Pharmacy & Health Sciences, Campbell University, Buies Creek, NC 27506, USA; ^3^Pharmacy Department, Durham Veterans Affairs Medical Center, Durham, NC 27705, USA; ^4^Department of Medicine, Duke University Medical Center, Durham, NC 27710, USA

## Abstract

*Introduction*. Hepatotoxicity is a concern in HIV/hepatitis C virus (HCV) coinfected patients due to their underlying liver disease. This study assessed the incidence of hepatotoxicity in HIV/HCV co-infected patients in two outpatient infectious diseases clinics. *Methods*. HIV/HCV co-infected adults were included in this retrospective study if they were PI or NNRTI naïve at their first clinic visit and were initiated on an NNRTI- and/or PI-based antiretroviral regimen. Patients were excluded if they had active or chronic hepatitis B virus (HBV). The primary objective was to determine the overall incidence of severe hepatotoxicity. *Results*. Fifty-six of the 544 patients identified met inclusion criteria. The incidence of severe hepatotoxicity was 10.7% (6/56 patients). Severe hepatotoxicity occurred with efavirenz (*N* = 2), nevirapine (*N* = 1), indinavir (*N* = 1), nelfinavir (*N* = 1), and saquinavir/ritonavir (*N* = 1). *Conclusion*. The incidence of severe hepatotoxicity appears to be low in this retrospective analysis of HIV/HCV co-infected patients receiving a PI-and/or NNRTI-based regimen.

## 1. Introduction

The advent of highly active antiretroviral therapy (HAART) in the treatment of human immunodeficiency virus (HIV) infection has significantly decreased the incidence of opportunistic infections as well as improved morbidity and mortality among HIV patients. However, along with these positive outcomes, HAART is associated with a host of adverse reactions such as hepatotoxicity, hyperlipidemia, hyperglycemia, and lactic acidosis. Hepatotoxicity can interrupt HIV therapy and cause an increase in morbidity and mortality [1]. Protease Inhibitors (PIs), nonnucleoside reverse transcriptase inhibitors (NNRTIs), and the chemokine receptor 5 (CCR5) antagonist, maraviroc, can be hepatotoxic with asymptomatic elevations in transaminase levels and, rarely, liver failure and death [2]. Approximately 1/3 of HIV infected patients in the United States are coinfected with HIV and hepatitis C (HCV) [3]. Often these patients have elevated liver function tests secondary to chronic HCV infection, which can be aggravated with the addition of hepatotoxic antiretrovirals for the treatment of their HIV infection. 

Several studies have evaluated hepatotoxicity associated with HAART in patients coinfected with HIV and either HCV or hepatitis B virus (HBV) [2–9]. HIV/HCV-co-infection has been estimated to increase the risk of HAART-associated hepatotoxicity 2- to 10-fold compared to HIV-monoinfection [4, 5]. Published retrospective studies evaluating the risk of developing severe hepatotoxicity with HAART in HIV/HCV-co-infected patients are limited by their short followup time of approximately one year. In addition, studies investigating exposure with newer protease inhibitors have not been performed. This study evaluated the effect of HAART and hepatotoxicity in HIV/HCV coinfected patients beginning with their initial visit at the Duke University Medical Center (DUMC) Infectious Diseases Clinic and the Durham Veterans Affairs Medical Center (DVAMC) Infectious Diseases Clinic between August 1, 1994 and December 31, 2006.

## 2. Methods

This was a retrospective study conducted at the DUMC and DVAMC Infectious Diseases Clinics and was approved by the Institutional Review Boards of both facilities with a waiver for patient consent. Medical and pharmacy records of patients were reviewed to identify eligible patients. Patients were included in the study if they met all of the following criteria: ≥18 years of age, HIV-infected with HCV coinfection, followed at either the DUMC or DVAMC Infectious Diseases Clinics from their initial visit between August 1, 1994 and December 31, 2006, PI or NNRTI naïve at their first clinic visit, received a PI- and/or NNRTI-based regimen during the study period, and had baseline liver function tests (LFTs) both prior to starting HAART and during treatment. Patients were excluded if they had active or chronic HBV. 

The following data was collected: demographic information, baseline liver enzymes (AST, ALT) prior to first antiretroviral therapy (ART) regimen and for subsequent regimens that the patient was changed to, subsequent liver enzymes during ART therapy for the study period, HCV genotype, baseline and subsequent PI/NNRTI regimens through December 31, 2006 and the duration of each regimen in months, data regarding rationale for changing PI or NNRTI regimens, plasma HIV-RNA level at baseline defined as viral load closest in time to the first HAART regimen, CD4 lymphocyte count at baseline defined as CD4 count closest in time to the first HAART regimen, HCV treatment at any time, alcohol consumption at baseline defined as no intake, moderate intake (not >1 drink/day), heavy intake (>1 drink/day), or unable to determine, and subsequent alcohol consumption was evaluated if LFT elevations were noted.

The primary endpoint of the study was to determine the incidence of severe hepatotoxicity (defined in accordance with AIDS Clinical Trials Group criteria as grades 3 or 4) in coinfected HIV/HCV patients receiving a PI- and/or NNRTI-based antiretroviral regimen [10]. In patients with normal baseline transaminases, grades 3 and 4 hepatotoxicity was defined as an alanine aminotransferase (ALT) and/or aspartate transaminase (AST) increase of 5.1–10 times the upper limits of normal (ULN) or >10 times the ULN, respectively. In patients with elevated baseline transaminases grades 3 and 4 hepatotoxicity was defined as an ALT and/or AST increased to 3.6–5 times the patient's baseline values or >5 times the patient's baseline, respectively. The incidence was expressed as the number of episodes of severe hepatotoxicity per person exposed during the study period. Secondary endpoints were to determine the incidence of severe hepatotoxicity among the individual PIs and NNRTIs as well as a class comparison (i.e., PIs versus NNRTIs). For both of the secondary objectives, the incidence was expressed as the number of severe hepatotoxic events per total months of exposure. To allow for comparison between individual drugs and class comparisons, the incidence of severe hepatotoxicity was normalized to 100 months of exposure. 

Data was entered into a Microsoft Access database (Office 2003, Microsoft Corporation Seattle, WA, USA) and analyzed using SAS (version 9.2, SAS Corporation, Cary, North Carolina). Descriptive statistics were used to analyze the demographics, HCV genotype, CD4 count, viral load, and incidence of severe hepatotoxicity. 

## 3. Results

A total of 544 HIV/HCV coinfected patients were identified, however, only 56 patients met all of the inclusion criteria. The primary reason patients were excluded from the study was that they were not treatment naïve at their first clinic visit (67.4%). Other reasons patients were excluded from the study included: active or chronic hepatitis B virus (21.5%), not receiving a PI- or NNRTI-based regimen (4.7%), insufficient data available, or not receiving antiretroviral therapy during the study period. Baseline demographics of the study population are described in Table 1. Patients were mostly male (75%) with a mean age of 50 years (±SD 7). A majority of patients were African-American (77%) with a baseline median CD4 count and viral load of 233 cells/mm^3^ (range: 5–960 cells/mm^3^) and 42,078 copies/mL (range: <100–799,059 copies/mL), respectively. There was insufficient information available to quantify baseline and subsequent alcohol consumption as well as receipt of HCV therapy.

The overall cumulative exposure of NNRTIs or PIs among study patients was 1203 and 996 months, respectively. The greatest exposure within the NNRTI class and PI class was with efavirenz (923 months) and lopinavir/ritonavir (269 months), respectively. Atazanavir/ritonavir was the PI with the second highest period of exposure of 213 months. For the primary endpoint, the incidence of severe hepatotoxicity (grades 3 or 4) associated with any PI- or NNRTI-based regimen was 10.7% (*N* = 6). Severe hepatotoxicity occurred with the following antiretrovirals: efavirenz (*N* = 2), nevirapine (*N* = 1), indinavir (*N* = 1), nelfinavir (*N* = 1), and saquinavir/ritonavir (*N* = 1) (Table 2). All of the hepatotoxic events were grade 3, except one grade 4 event with nevirapine. For secondary endpoints, the normalized incidence of severe hepatotoxicity was the highest with indinavir (1.03 cases/100 months of exposure) (Table 2). Notably, no cases of severe hepatotoxicity were observed with lopinavir/ritonavir or atazanavir/ritonavir, which had the greatest months of exposure among PIs. The normalized incidence of severe hepatotoxicity for NNRTIs was 0.249 cases/100 months of exposure and 0.301 cases/100 months of exposure for PIs. All cases of hepatotoxicity occurred in separate individuals. Five out of the 6 cases of hepatotoxicity occurred on the first regimen the patient was prescribed. The time between starting antiretroviral therapy and the development of hepatotoxicity ranged from 3 weeks to 18 months. Four of the 6 patients changed regimens and subsequently had normalization of their enzymes. Two patients remained on the same regimen until the end of the study without normalization of their enzymes.

Rationale for changing or discontinuing an NNRTI- or PI-based regimen was identified in 33 cases. The most common reason for changing a regimen was an adverse effect related to the medication (40%), resistance/treatment failure (21%), nonadherence (18%), regimen simplification (15%), and hepatotoxicity (6%).

## 4. Discussion

HIV patients coinfected with HCV represent about a third of the HIV population in the US and are at a greater risk for liver toxicity with the use of potentially hepatotoxic antiretrovirals [6]. Questions regarding the safest antiretrovirals to use in this patient population have been investigated in several published clinical trials; however, many of these studies included patients who were also infected with HBV. For example, in a prospective cohort study of HIV patients coinfected with either HBV or HCV (*N* = 298), hepatotoxicity was assessed in patients who initiated either a PI-based (*n* = 211) or dual nucleoside analog (*n* = 87) antiretroviral regimen [4]. Forty-eight percent of patients in the study on a PI-based regimen were coinfected with HCV, and were followed for a median of approximately 6 months. Less than 3% of patients in the study were coinfected with HBV. Overall, the incidence of severe hepatotoxicity (grade 3 or 4 as defined by the AIDS Clinical Trials Group) in the entire study cohort (irregardless of hepatitis coinfection) was 10.4%. Among HBV or HCV coinfected patients, severe hepatotoxicity (grade 3 or 4) occurred in 12%. Regimens that included ritonavir were associated with a greater incidence of hepatotoxicity than other regimens, but appeared to be unaffected by HCV/HBV coinfection. In a multivariate model, use of ritonavir, but not HBV/HCV coinfection status, was independently associated with severe hepatotoxicity in the cohort (adjusted OR 8.6; 95% CI 3-24.6). Among those receiving nonritonavir based-regimens, there was a higher incidence of hepatotoxicity in those with HCV or HBV infection compared to those without coinfection (9.4% versus 2.7% resp., RR 3.7 95% CI 1.0–11.8) [4]. Lai et al. found that 33% of HIV/HCV coinfected patients compared to 12% of noncoinfected patients experienced severe hepatotoxicity on HAART [6]. Co-infected patients also developed liver enzyme elevations more rapidly than noncoinfected patients (*P* = .0001). On multivariate analysis, coinfection with HBV or HCV was associated with severe hepatotoxicity (*P* = .001), but there was no association with individual NRTI or PI medications possibly due to small sample size [6]. A larger study published in 2002 by Sulkowski et al. found a significantly greater risk of severe hepatotoxicity in coinfected patients receiving nevirapine or efavirenz-based regimens compared to HCV-uninfected persons [9]. In the HCV infected group, 19 of 124 (15.3%) patients on efavirenz based regimens experienced severe hepatotoxicity compared to 6 of 188 (3.2%) uninfected HCV patients. A majority of patients in the study received concurrent protease inhibitors and severe hepatotoxicity was the highest among this group of patients; however, the risk persisted irrespective of concomitant protease inhibitor use [9]. 

Overall, our finding of an incidence of severe hepatotoxicity in coinfected patients of 10.7% is similar to the rate of severe hepatotoxicity related to HAART in noncoinfected patients in these studies. In our study we found cases of hepatotoxicity occurred with older, less commonly used PIs (saquinavir, indinavir, and nelfinavir). Interestingly, when reviewing newer agents such as lopinavir/ritonavir and atazanavir/ritonavir which had the largest months of exposure for the PIs, no severe hepatotoxic events were identified. This may suggest that these newer PIs may be relatively safe to use in this patient population. The commonly used NNRTI, efavirenz, was associated with two cases of severe hepatotoxicity; however, this drug had the most months of usage which led to a low normalized incidence.

A major limitation of our study is the small sample size since the patients were used as their own controls. Future studies could include a control group of noncoinfected patients so patients that are treatment experienced could be included. Additionally, LFT changes documented in the study could have been attributed to other causes such as HCV or the use of other concomitant hepatotoxic drugs or substances, such as alcohol. In a retrospective study assessing severe hepatic injury after beginning HAART, coinfected patients had a 3.99 greater chance of developing severely elevated transaminase levels compared with patients not coinfected with HCV. This risk was especially greater in coinfected patients with identified alcohol abuse [8]. Although we attempted to include information regarding alcohol intake, it was difficult to assess this in a retrospective study due to poor documentation in the medical records. Information regarding current use of injection or illicit drugs that could potentially contribute to the risk of hepatotoxicity was not collected. Adherence to HAART was difficult to verify in patients followed at the DUMC Infectious Diseases Clinic since it was not always documented in the patient's clinic notes nor did the facility dispense the medications to their patients as is routinely done at the DVAMC. Risk estimates could be higher for those with better adherence and underestimated for those with poor adherence. Finally, due to the low incidences, we had insufficient power to compare different drugs or classes with one another. 

In conclusion, additional studies are needed to gain a better appreciation for the incidence of severe hepatotoxicity in HIV patients coinfected with HCV. However, based on this retrospective analysis, the incidence of severe hepatotoxicity appears to be relatively low in HIV/HCV-coinfected patients receiving either a PI- or NNRTI-based regimen.

## Figures and Tables

**Table 1:** Baseline Demographics (*N* = 56).

Mean age (range)	50 years (30–62)
Sex	
Male	42 (75%)
Female	14 (25%)
Race	
African-American	43 (77%)
Caucasian	7 (13%)
Unknown	4 (6%)
Hispanic	1 (2%)
Hawaiian	1 (2%)
HCV genotype	
1	31 (55%)
2	2 (4%)
Unknown	23 (41%)
Median baseline CD4 count (range)	233 cells/mm^3^ (5–960)
Median baseline viral load (range)	42,078 copies/mL (<100–799,059)
Median baseline AST (range)	66 U/L (17–305)
Median baseline ALT (range)	56 U/L (10–259)

**Table 2:** Primary and secondary endpoints.

Primary endpoint:	
Incidence of severe hepatotoxicity	10.7% (6/56 Patients)
Secondary endpoints	
NNRTIs	3 cases/1203 months
	0.249 cases per 100 months of exposure
Efavirenz	2 cases/923 months
	0.217 cases per 100 months of exposure
Nevirapine	1 case/280 months
	0.357 cases per 100 months of exposure
PIs	3 cases/996 months
	0.301 cases per 100 months of exposure
Indinavir	1 case/97 months
	1.03 cases per 100 months of exposure
Nelfinavir	1 case/169 months
	0.592 cases per 100 months of exposure
Saquinavir/Ritonavir	1 case/100 months
	1.00 cases per 100 months of exposure
